# Low-Temperature Superplasticity of Ultrafine-Grained Aluminum Alloys: Recent Discoveries and Innovative Potential

**DOI:** 10.3390/ma17133311

**Published:** 2024-07-04

**Authors:** Elena V. Bobruk, Nail G. Zaripov, Ilnar A. Ramazanov, Nguyen Q. Chinh, Ruslan Z. Valiev

**Affiliations:** 1 Institute of Physics of Advanced Materials, Ufa University of Science and Technology, Ufa 450076, Russia; 2 Department of Materials Physics, Eötvös Loránd University, 1117 Budapest, Hungary; chinh@metal.elte.hu; 3 Laboratory for Dynamics and Extreme Performance of Advanced Nanostructured Materials, Saint Petersburg State University, Saint Petersburg 198504, Russia

**Keywords:** low-temperature superplasticity, ultrafine-grained structure, grain boundary, segregation, mechanical property

## Abstract

The last two decades have witnessed significant progress in the development of severe plastic deformation techniques to produce ultrafine-grained materials with new and superior properties. This review examines works and achievements related to the low-temperature superplasticity of ultrafine-grained aluminum alloys. The examples are provided of the possibility to observe low-temperature superplasticity in aluminum alloys at temperatures less than 0.5 T_melt_ and even at room temperature, and herein, we demonstrate the cases of achieving high ductility and high strength in aluminum alloys from processing utilizing severe plastic deformation. Special emphasis is placed on recent studies of the formation of segregations of alloying elements at grain boundaries in UFG Al alloys and their influence on the development of grain boundary sliding and manifestation of low-temperature superplasticity. In addition, the current status and innovative potential of low-temperature superplasticity in aluminum alloys are observed.

## 1. Introduction

The term “superplasticity” (SP) refers to the ability of polycrystalline materials to undergo abnormally high plastic deformation at relatively low values of yield stress under conditions of their high strain rate sensitivity and without noticeable strain hardening [1,2,3]. During tensile deformation, superplasticity conventionally manifests itself in specimen elongations up to 400% or more. Numerous studies have shown that in the presence of a certain microstructure, temperature, and strain rate, superplasticity is a universal state of materials and occurs not only in metallic materials, but also in intermetallic compounds, composites, and ceramics [1,2,3,4,5,6]. A fine-grained microstructure, elevated deformation temperature, and low deformation rates are the main conditions for the manifestation of such structural superplasticity. At the same time, the grain size of these materials is typically at the micron level (usually about or somewhat less than 10 μm), the deformation temperature during SP is usually above 0.6–0.8 T_melt_ (where T_melt_—homologous melting point of the material), and strain rates are in the range from 1 × 10^−5^ s^−1^ and 1×10^−3^ s^−1^.

Superplastic behavior is usually described by a relation in the form of the basic equation:

(1) $$\overset{•}{\varepsilon }=A\frac{D\cdot G\cdot b}{K\cdot T}{\left(\frac{b}{d}\right)}^{p}{\left(\frac{\sigma }{G}\right)}^{n}$$

where *D* is the diffusion coefficient, *G* is the shear modulus, *b* is the Burgers vector, *K* is Boltzmann constant, *T* is absolute temperature, *d* is average grain size, σ is flow stress, *p* is the exponent usually equal to 2, *n* is the reciprocal of the strain rate sensitivity of the stress *m*, and *A* is nondimensional coefficient.

This equation demonstrates an important connection between the external deformation characteristics and the internal structure of materials. It follows that a decrease in grain size should lead to both an enhancement in the strain rate at which superplastic behavior is observed and a decrease in the temperature of superplastic deformation, which is attributed to the physical nature of SP deformation, where grain boundary sliding (GBS) represents the primary deformation mechanism. Its contribution to the deformation process is observed to increase with decreasing grain size.

In this regard, the first works on the production of ultrafine-grained (UFG) metals and alloys using severe plastic deformation techniques, which proved the possibility of reducing the SP temperature to 0.6 T_melt_ and below, aroused great interest among the experts dealing with superplasticity [7,8]. To obtain UFG structures with a grain size less than 1 μm, the facilities for high-pressure torsion (HPT) and equal channel angular pressing/extrusion (ECAP/ECAE) were used in these works for the first time, and later, these techniques became the most popular methods of severe plastic deformation (SPD) for the production of UFG materials [9].

**Figure 1:**
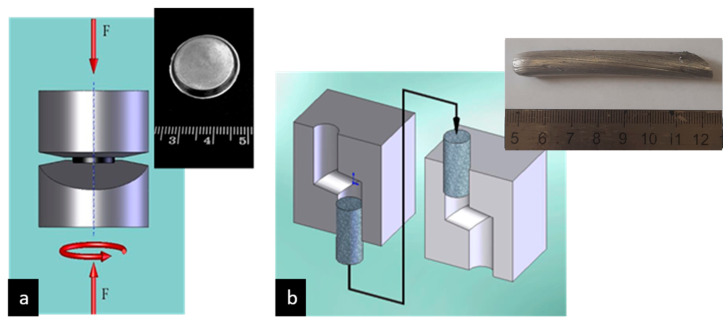
(**a**) A schematic illustration of HPT assembly of two anvils and a thin sample (an insert); (**b**) a schematic illustration of ECAP device and a billet after processing (an insert). Adapted with permission from [9]. Copyright 2014, The Minerals, Metals & Materials Society.

HPT is a processing technique with severe plastic deformation, in which billets, typically shaped as disks with a diameter of 10–20 mm and a thickness of up to 2 mm, are subjected to shear deformation by means of torsion under conditions of high applied hydrostatic pressure (up to 10 GPa) to form an UFG structure. The workpiece is placed on flat anvils (Figure 1a) or within a cavity in the anvils, and a pressure of 1 to 10 GPa is applied to it. The sample is plastically deformed by torsion through the rotation of one of the anvils.

Another effective SPD technique that has been demonstrated to produce the UFG structure in bulk billets of metals and alloys is ECAP with parallel channels (Figure 1b), which comprises the deformation of billets through shear in the intersection zone of channels of equal cross-section, typically at an angle of 90° [9]. The advantage of this method is that the workpiece can be subjected to significant shear deformation without a corresponding change in its dimensions. The process of repeated pressing cycles leads to the formation of a homogeneous UFG structure in the material.

To date, a number of SPD techniques have been developed for the purpose of grain refinement in a variety of metallic materials [10,11]. In terms of research on superplasticity in Al alloys with ultrafine grains, along with HPT and ECAP methods, accumulative rolling (ARB) and friction stir processing (FSP) techniques have gained popularity [12,13,14]. A review of the influence of ultrafine grain size on the manifestation of superplasticity in a number of alloys can be found in recent publications [15,16,17].

At the same time, in accordance with Equation (1), the SP behavior is also related to diffusion along the grain boundaries, which regulates the grain boundary sliding rate. Consequently, the rate of grain boundary diffusion depends on the structural and compositional characteristics of grain boundaries, including their morphology, the presence of dislocations, and the segregation of alloying elements. This approach is of particular importance for the UFG materials processed by SPD techniques, since the grain boundaries formed during such processing have a number of structural features, including defect structure and segregations. As is shown herein, these features often play a determining role in the manifestation of low-temperature superplasticity of UFG materials.

It should also be noted that the SP state is favorable for the fabrication of complex products from low-plastic materials. However, high temperatures and low strain rates hinder the wide application of this phenomenon. In this regard, the manifestation of SP at reduced temperatures opens up opportunities for new innovative developments in the field of processing of hard-to-deform materials and their forming.

In this review, the latest achievements in the field of low-temperature superplasticity, established on a number of model and industrial Al-based alloys, the general patterns of manifestation of low-temperature superplasticity and the innovative potential of these developments are investigated. A particular focus is placed on establishing the physical characteristics of this phenomenon towards the materials processed by severe plastic deformation (SPD), where the ultrafine grain size as well as the state of grain boundaries related to their structure and the presence of grain boundary segregations also play a pivotal role.

## 2. Phenomenon of Superplasticity in Al Alloys

Superplastic aluminum alloys usually include alloys of the Al-Mg-Li, Al-Zn-Mg-Cu (7000 series), Al-Zn, Al-Mg, Al-Mg-Sc (5000 series), and Al-Cu-Mg-Mn (2000 series) systems where microstructure refinement is conventionally obtained through deformation by rolling or extrusion and heat treatment [2,3]. Such processing of Al alloys using rolling or extrusion with subsequent annealing typically results in the formation of grains with a size of approximately 10 µm. Consequently, these alloys exhibit superplastic behavior at strain rates of 10⁻^4^ to 10⁻^3^ s^−1^ and temperatures of 450 to 500 °C (approximately 0.69–0.76 T_melt_). Table 1 illustrates the superplastic behavior of a number of commercial Al alloys with ultrafine grains in the submicron range. As is observed herein, the temperature at which superplasticity is manifested is considerably lower, ranging from 250 to 350 °C [12,13,14,15,18,19,20,21,22].

At the same time, the majority of works on low-temperature superplasticity consider only the influence of grain sizes in terms of temperature and processing techniques (16, 17). However, they do not address the results of studies on the special state of high-angle boundaries that are formed during SPD processing. This includes the formation of non-equilibrium grain boundaries and segregation of alloying elements in them. As was mentioned before, grain size is not the sole determining factor in the transition of alloys to low-temperature superplasticity.

**Figure 2:**
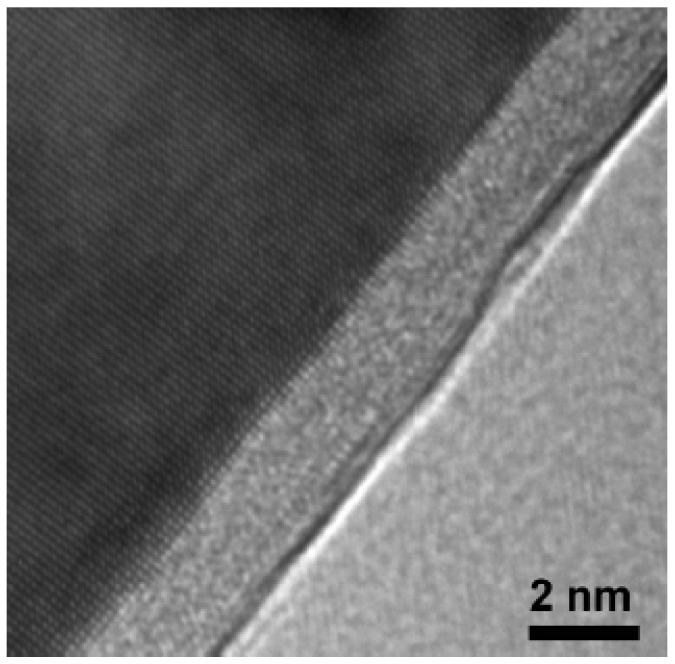
High-resolution transmission electron microscopy image demonstrating a wetting Zn layer along Al/Al grain boundary in the UFG Al-30Zn sample. Reproduced with permission [25]. Copyright 2006, Elsevier.

**Figure 3:**
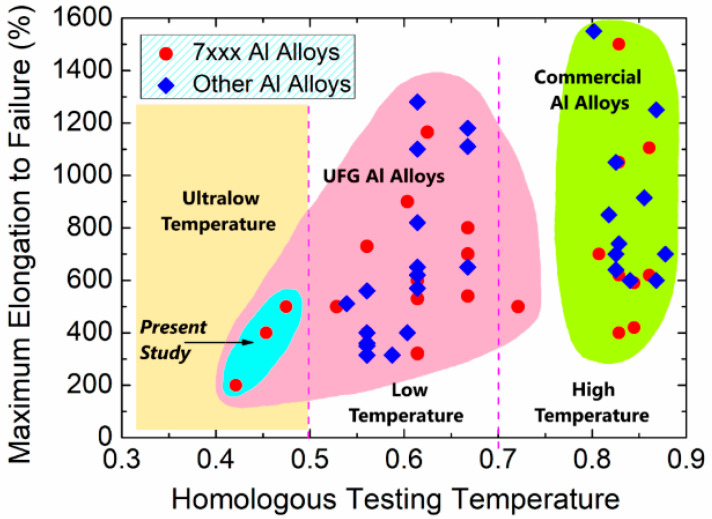
Parameters of low-temperatures superplasticity in Al alloys of 7000 (AlZnMg-based) and other systems (AlMg- and AlLi-based). Reproduced with permission [23]. Copyright 2021, Taylor & Francis.

The first evidence of this phenomenon was observed in [24] on an Al-30% Zn alloy with a grain size of 500 nm, obtained after 10 revolutions of high-pressure torsion (HPT). This alloy exhibited unusually high ductility at room temperature, with a maximum elongation of 235% and a strain rate sensitivity, m, of 0.31 at a strain rate of 10^–4^ s^–1^. The results demonstrated that the occurrence of these characteristics of SP at room temperature relates not only to the formation of an UFG structure in the alloy, but also to the presence of segregations of Zn atoms at grain boundaries. The segregation of Zn solutes is shown in the high-resolution transmission electron microscopy image in Figure 2, where a Zn layer with a thickness of about 2 nm can be seen along an Al/Al grain boundary in the UFG Al-30Zn sample [25]. The results of calculations have shown the fast diffusion of Zn atoms in aluminum grain boundaries and thus the advantage of such grain boundaries in the low-temperature grain boundary sliding mechanism. The segregation of Zn solute atoms has been identified as a contributing factor to the active grain boundary sliding observed not only in the model alloys of the Al-Zn system but also in commercial alloys of the Al-Zn-Mg system (Figure 3) [23].

A subsequent series of studies on model alloys of the Al-Zn system [26,27], and also on commercial alloys of the Al-Zn-Cu system [23,28], demonstrated the potential for achieving low-temperature superplasticity in these materials at temperatures below 200 °C, even at room temperature. In all cases, grain boundaries were observed in a nonequilibrium state and containing significant zinc segregations, which is important for understanding the deformation mechanisms of their SP behavior.

## 3. Physical Nature and Deformation Mechanisms of Low-Temperature Superplasticity

**Figure 4:**
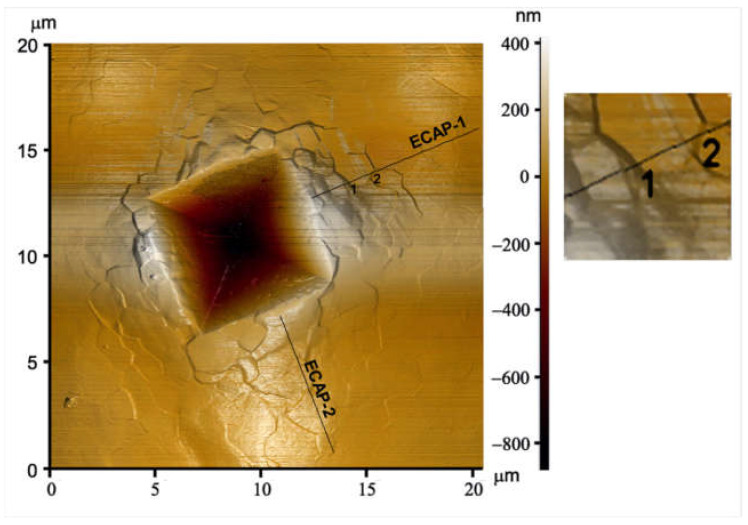
Atomic force microscopy image revealing intensive grain boundary sliding in an ultrafine-grained pure aluminum sample. Reproduced with permission [29]. Copyright 2006, Elsevier.

Numerous experiments demonstrated that the formation of ultrafine grains increases the contribution of grain boundary sliding to deformation, and in many cases, this occurs even at low temperatures. For example, Figure 4 shows an atomic force microscopy (AFM) image demonstrating grain boundary sliding at room temperature in an ECAP-processed UFG pure Al sample with an average grain size of about 1 µm [29]. The three-dimensional AFM image was taken on the morphology of the surface of UFG sample deformed by Vickers indenter. As is seen, the morphology of the surface around the Vickers pattern with the size of about 8 µm reflects unambiguously the displacement of fine grains relatively to each other, demonstrating visibly the occurrence of grain boundary sliding in the UFG matrix.

However, despite visible grain boundary sliding, the ductility of this UFG Al remains poor at room temperature. This is because in these cases, the grain boundary diffusion at low temperatures is not fast enough to enhance the grain boundary sliding. Such UFG materials can generally be characterized by a low strain rate sensitivity, m, which is only around 0.1 [29].

To determine the patterns and nature of low-temperature superplasticity in the UFG Al-30%Zn alloy, a new experiment was recently conducted in situ in an electron microscope column using tensile straining [27]. This was followed by precision identification of grain boundaries. The observations demonstrated that SP is achieved through grain boundary sliding and rotational deformation modes, which are facilitated by the continuous diffusion of Zn atoms from the grain body to the grain boundaries. This leads to the formation of a Zn nanolayer at the boundaries, which serves as a “lubricant” to reduce the energy barrier for grain boundary sliding (GBS).

**Figure 5:**
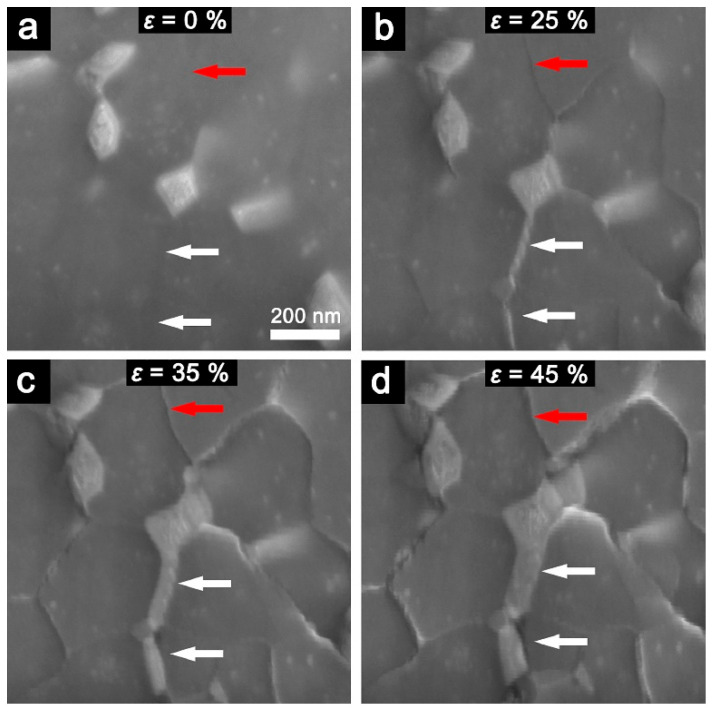
Microstructure in backscattered electrons during in situ tensile deformation by (**a**) 0%, (**b**) 25%, (**c**) 35%, and (**d**) 45%. Red and white arrows mark the positions of grain boundaries. Areas with light contrast Zn-enriched zones. Reproduced with permission [27]. Copyright 2023, Elsevier.

Figure 5 presents the evolution of the microstructure and the distribution of Zn atoms inside the grain body and at the boundary during tensile straining. Before straining (Figure 5a), relatively large areas with light contrast were visible mainly at the triple junctions of grain boundaries, with small light spots visible inside the grains. It can be seen that the Zn atom-enriched regions were mainly located at some triple junctions and were sparsely dispersed within the Al-enriched grains. The boundary region, indicated by two arrows, did not show clear contrast before tensile straining. However, as shown in Figure 5b, clear light contrast lines were observed in the region of grain boundaries after straining by 25%. The change in contrast suggests Zn segregation at the grain boundary. Further, the width of the white region increased up to 50 nm in Figure 5c and up to 100 nm in Figure 5d. The phenomenon shown in Figure 5 can be considered to be the growth of zinc grains at grain boundaries provided that aluminum is poorly soluble in zinc grains. The gaps between adjacent grains formed during plastic deformation, as illustrated by the area marked with red arrows, demonstrate that the GBS occurred simultaneously with the separation of zinc.

**Figure 6:**
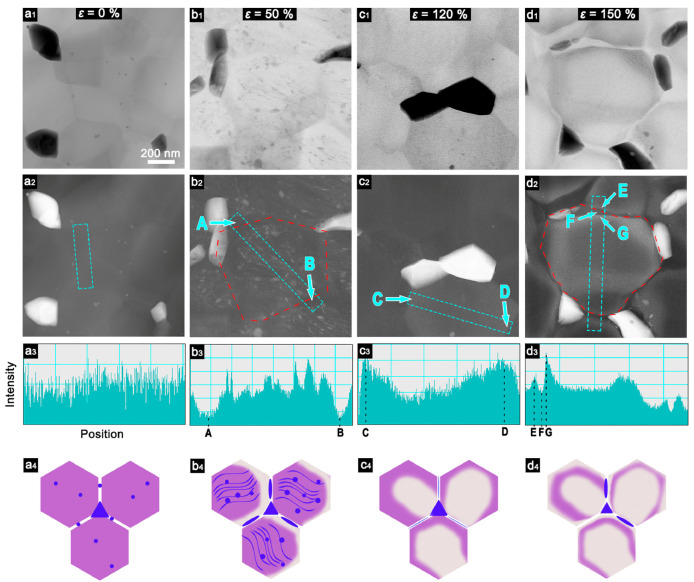
Each column includes an annular bright-field STEM image (**a1**–**d1**) and a STEM-HAADF image (**a2**–**d2**) of the same area, an image of the intensity distribution along the narrow rectangular region indicated by the blue dotted lines in the STEM-HAADF image (**a3**–**d3**), and also a schematic representation of the distribution of zinc in the sample at a deformation of (**a**) 0%, (**b**) 50%, (**c**) 120%, and (**d**) 150% (**a4**–**d4**). The letters A–G indicate the sample positions in the second and third rows. Red dotted lines indicate grain boundaries. In the schematic diagrams, blue, purple, and white regions represent Zn, Al/Zn mixed regions, and Zn-depleted regions, respectively. Reproduced with permission [27]. Copyright 2023, Elsevier.

Prior to tensile straining, the microstructure comprised three distinct types of Zn atoms: (1) coarse Zn grains with an HCP lattice at the triple junctions of the matrix grains; (2) in supersaturated solid solution of the aluminum matrix; (3) nanoscale Zn precipitations segregated within the aluminum grains (Figure 6(a1–a4)). The image contrast is relatively uniform across the grains with the exception of the dispersed nanoscale Zn precipitations within the matrix grains, indicating that the distribution of Zn within the aluminum matrix was homogeneous. Figure 6(b1–b4) demonstrates a redistribution of Zn observed at 50% straining, resulting in two phenomena: (1) the formation of Zn-depleted zones in close proximity to the boundary (intensity decreases at points A and B), likely due to the diffusion of Zn to neighboring boundaries, and (2) the dynamic precipitation of a high density of nanoscale Zn particles within the grains. The presence of numerous light lines connecting these nanoscale Zn precipitations (Figure 6(b2)) indicates the occurrence of preferential segregation and precipitations along these connecting lines, which may be dislocation lines. However, it is noteworthy that plastic deformation mostly occurred due to grain rotation and GBS.

At 120% strain, Figure 6(c2) and the corresponding intensity profile in Figure 6(c3) demonstrate the disappearance of the nanoscale precipitates, the transition of the centre of individual grains to the darkest region, and the emergence of lighter contrast in the regions close to the boundaries. Evident peaks at points C and D along with a minimum in the center of the intensity profile are observed providing compelling evidence of Zn diffusion during deformation. It is noteworthy that the reduction in Zn concentration occurs in a sequential manner, from the center to the boundaries. This is evidenced by the distribution of Zn, which exhibits a minimum in the grains. The schematic diagram also illustrates this process, with the violet area moving from the center to the boundaries (Figure 6(a4–d4)). Zn atoms in enriched zones near grain boundaries appear both from the center of matrix grains and from Zn grains at the boundaries due to the diffusion process.

The images obtained at 150% strain demonstrate a typical layered structure, characterized by a dark contrast at the boundary and a lighter contrast in the adjacent region. This is illustrated in Figure 6(c2). This phenomenon is also evident in the intensity profile presented in Figure 6(d3).

These studies have advanced our understanding of the diffusion-stimulated mechanism of SP deformation, which is a prerequisite for the development of new materials that exhibit low-temperature superplasticity at room temperature.

**Figure 7:**
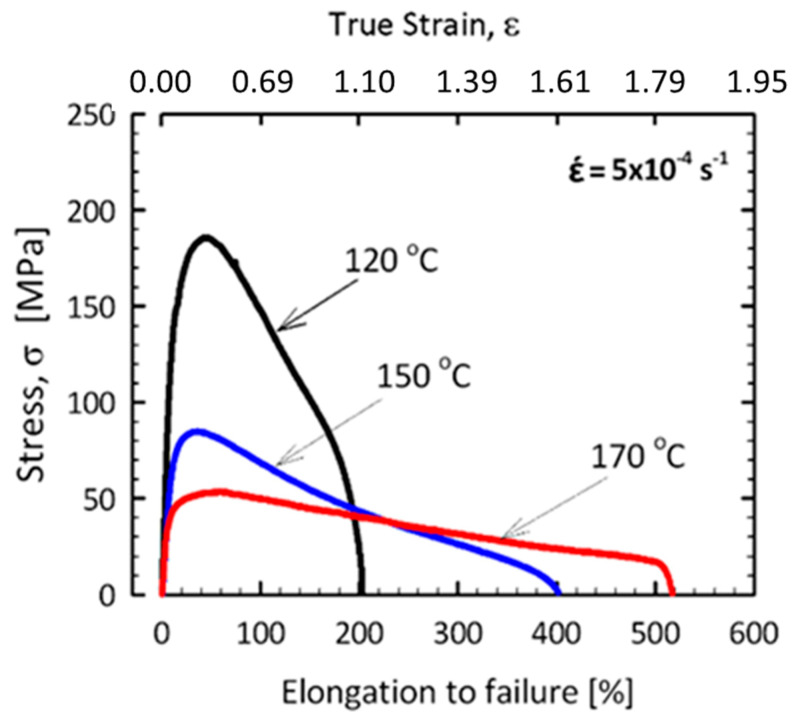
Stress–strain (σ–ε) curves, showing a total elongation higher than 500% at 170 °C (0.47 homologous temperature) for the UFG Al-Zn-Mg alloy superplastically deformed at 170 °C and a strain rate of 5 × 10^−4^ s^−1^. Reproduced with permission [23]. Copyright 2021, Taylor & Francis.

Similar patterns were observed on the example of the commercial alloy of the Al-Zn-Mg system processed by HPT at RT for ten revolutions. Ultrafine-grained structure with a grain size of 170 nm and a regulated distribution of secondary phase MgZn_2_ was formed. This was the first time it was shown that it is possible to achieve a record level of superplasticity with elongation higher than 500% at very low temperatures under 0.47 T_melt_, as shown in Figure 7. The record elongation is due to the formation of UFG structure with a grain size of 170 nm and predominantly high-angle grain boundaries containing grain boundary segregations of alloying elements in the form of interlayers and nanostructured second phase precipitations in the grain body [23].

**Figure 8:**
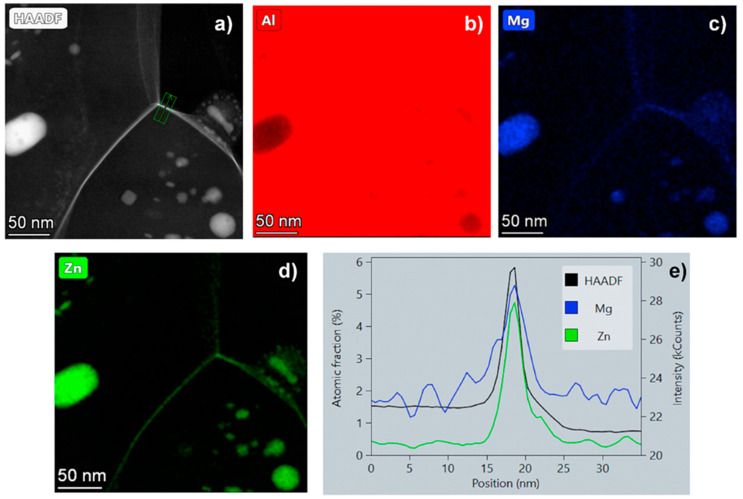
Typical structure of grain boundaries in the UFG AlZnMg sample superplastically deformed at 170 °C, presented by (**a**) high-magnification HAADF image showing Zn-rich boundaries, (**b**–**d**) corresponding elemental maps for Al, Mg, and Zn, respectively, and (**e**) EDS profiles across a boundary (along the green arrow in image (**a**)), showing the segregation of Zn and Mg solute atoms into Al/Al grain boundaries in the UFG matrix. Reproduced with permission [23]. Copyright 2021, Taylor & Francis.

The key point in the mentioned record low-temperature superplasticity is the segregation of the solute atoms into grain boundaries of the UFG structure during the decomposition due to SPD processing. Experimental results [23] have shown unusually high strain rate sensitivity (m≈0.43) and low activation energy (≈68 kJ/mole) for the mentioned superplastic deformation. Calculations using this activation energy revealed that the diffusion coefficient, *D* characterizing the GBS at 170 °C of this sample, is about two orders of magnitude higher than that estimated for GBS in pure aluminum. Thanks to sufficiently fast diffusion, grain boundary sliding can last longer without crack formation, leading to the record elongation. The fast diffusion is the consequence of the unique structure of the grain boundaries segregated by both Zn and Mg solute atoms, which both decrease the melting point of the grain boundary phase of the UFG material, increasing the homologous testing temperature, and then leading to easier diffusion along grain boundaries [23]. Figure 8 presents a typical STEM-HAADF image and the corresponding element-map and profiles on the grain boundary structure of the superplastically deformed AlZnMg sample. It can be seen that in addition to the ${MgZn}_{2}$ phase particles, which appear as bright areas in the image due to the higher atomic number of Zn compared to aluminum, Zn-rich Al/Al grain boundaries are easily visibly observed (see Figure 8a). The corresponding element map reveal clearly the depletion of Al (Figure 8b), as well as the excess of Mg (Figure 8c) and Zn atoms (Figure 8d) in the Al/Al grain boundaries. The data obtained by element analysis presented in Figure 8e indicate the strong segregation of both solutes by showing about 4-times higher Mg and 10-times high Zn concentration inside the grain boundaries than that in the neighboring areas. It should be noted that although Mg segregation also has the mentioned decreasing effect in the local melting point of the grain boundary phase, the diffusion of Mg is much more difficult than the diffusion of Zn in the grain boundaries. Thus, the grain boundary sliding process is mainly controlled by the grain boundary diffusion of Zn atoms [23].

**Figure 9:**
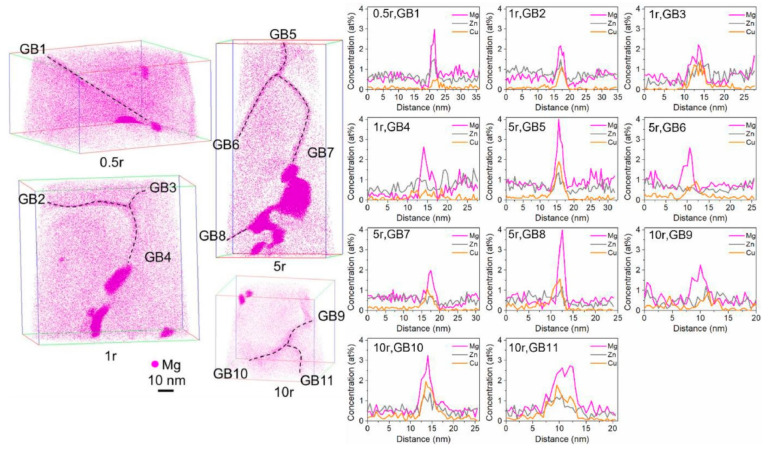
Three-dimensional reconstructed volumes of AA7075 processed by 2nd-stage HPT at 100 °C, with GBs and corresponding 1D concentration profiles along 11 marked GBs measured by using an analysis box with its z axis parallel to each GB normal. Reproduced with permission [30]. Copyright 2022, Elsevier.

The redistribution of alloying elements is clearly illustrated by atomic maps of 7075 alloy samples processed by two-stage HPT at RT, with marked GBs and corresponding 1D concentration profiles shown in Figure 9. The magnesium and copper contents of the twelve marked boundaries were in the range of 0.57–2.22 Mg atoms/nm^2^ and 0.52–1.82 Cu atoms/nm^2^. In boundary 4, no Zn segregation was observed, while in the others, clear Zn segregation was observed in the range of 0.03–1.67 Zn atoms/nm^2^ [30].

**Figure 10:**
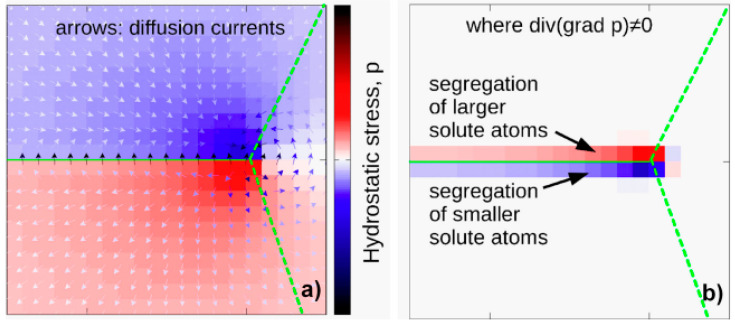
Trapping effect of sliding grain boundary. (**a**) Calculated hydrostatic stress component (*p*) around a slipped grain boundary (the arrows show the direction of the diffusion currents). (**b**) Accumulation points for solute atoms smaller (red) and larger (blue) than the matrix atoms (Al). The green dashed lines represent the grain boundaries. Reproduced with permission [23]. Copyright 2021, Taylor & Francis.

The results of the energy-dispersive analysis are presented schematically in Figure 9, which demonstrates the depletion of the matrix Al phase and the presence of an excess of Mg and Zn atoms at the grain boundaries of the Al/Al in ultrafine-grained sample. Measurements in different regions indicate that the proportions of zinc and magnesium vary along grain boundaries. In some regions, Zn atoms are more prevalent than Mg atoms at the Al/Al grain boundaries, as compared to other regions with the concentration of Mg being higher than that of Zn atoms. The matrix grain boundaries in this sample can be considered to exhibit high Zn and/or Mg concentrations. In accordance with the results of computer calculations, the formation of segregations at grain boundaries can be considered to be the decomposition of dispersed particles at moving grain boundaries. Figure 10 illustrates the effect of a sliding boundary on the trapping of larger (e.g., Mg) and smaller (e.g., Zn) solute atoms (in comparison to the Al matrix) on opposing sides of the sliding grain boundary.

These studies have enabled the first demonstration that in high-strength Al-Zn-Mg and Al-Zn-Mg-Cu alloys, the formation of ultrafine-grains through thermomechanical treatment, including the use of severe plastic deformation techniques, can result in the achievement of low-temperature superplasticity at temperatures below 200 °C (0.5 T_melt_), which is associated with the formation of Zn segregations at grain boundaries.

In the UFG alloys 1421 and 1570 of the Al-Mg system, evidence of superplasticity was obtained in [31]. Submicrocrystalline and nanocrystalline structures with grain sizes in the range of 100–400 nm were formed using HPT at RT, 10 turns and ECAP at 200 °C, 8 passes, and the ***Bc*** route. The maximum elongations were obtained over 1000% at a strain temperature of 350 °C, a strain rate of 10^−2^ s^−1^, and with elongations exceeding 300% at 200 °C and a strain rate of 10^−1^ s^−1^ [31]. As was demonstrated, the primary contributor to the sample deformation is cooperative grain boundary sliding along the boundaries of fragmentary groups of grains.

Further studies of UFG Al-Mg alloys showed the presence of segregations of Mg atoms along grain boundaries [21]. The addition of Sc and/or Zr to SPD-produced Al-Mg alloys is a common practice contributing to the formation of stable ultrafine-grained structures. This is due to the fact that the coherent nanoscale Al_3_Sc or Al_3_(Sc,Zr) precipitates formed during severe plastic deformation allow for efficient restraint of grain growth at elevated temperatures. This phenomenon has been observed and documented in numerous studies, as referenced in [32,33,34,35,36,37,38]. In alloys of the Al-Mg system processed by SPD, the grain boundaries of ultrafine grains typically exhibit a nonequilibrium configuration that is quiet unstable.

**Figure 11:**
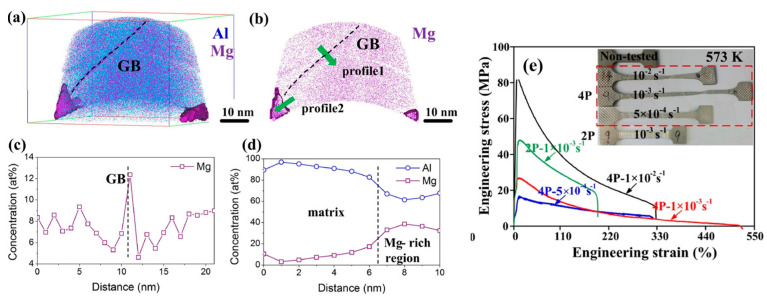
APT analyses of the 4P ECAPed Al–7Mg sample: (**a**,**b**) element distribution maps, where Al (blue) and Mg (pink) atoms are clearly shown; (**c**,**d**) the concentration profile of Mg solute across the GB and local strong Mg-rich region along the GB, respectively, indicated by green arrows in (**b**), confirming the heterogeneous distribution of Mg atoms along the GB; (**e**) tensile engineering stress–strain curves of the 2P and 4P Al–7Mg samples tested at various strain rates. Reproduced with permission [21]. Copyright 2021, Elsevier.

However, it has been demonstrated that nonequilibrium boundaries in ultrafine-grained alloys are preferred sites for the formation of solute segregations and nanoclusters [39]. In [21], the Al-7Mg alloy (wt.%) was subjected to six cycles of ECAP at room temperature, six passes, which resulted in the formation of an UFG structure with a grain size of approximately 500 nm and predominantly high-angle grain boundaries (volume fraction was 57%), which contain Mg segregations and β-phase Al_3_Mg_2_ particles (Figure 11).

The formation of such a structure allowed the achievement of high values of elongation, exceeding 500%, and strain rate sensitivity coefficient of m = 0.75 at 300 °C and a strain rate of 10^−3^ s^−1^ [21].

Additionally, in [21], the change in the UFG structure during low-temperature SP deformation was investigated. The authors concluded that grain boundary sliding plays a dominant role in the SP process, as evidenced by the sharp blurring of the texture and the high value of m~0.75. The calculated strain activation energy of ~78 kJ/mol is comparable to the self-diffusion energy of grain boundaries in Al alloys, further confirming the pivotal role of grain boundary sliding. Concurrently, the presence of Mg segregations and nanoscale Al_3_Mg_2_ phase precipitates impedes grain boundary migration and constrains grain growth.

Thus, to date, numerous studies have demonstrated that the manifestation of low-temperature superplasticity of Al alloys is due not only to the formation of an ultrafine-grained structure with nanoscale grain sizes by SPD techniques, but also to the formation of grain boundary segregations and clusters of various alloying elements. The abovementioned studies provide compelling evidence that Zn segregation promotes the diffusion and development of GBS. However, evidently, further research is required to clarify the question of high priority related to low-temperature superplasticity, particularly the role of different element segregations at grain boundaries in its manifestation.

## 4. Summary and Conclusions

This article demonstrates that the use of various SPD techniques to obtain ultrafine-grained metals and alloys enables the control of their superplastic behavior, ensuring the manifestation of the effect at low temperatures, i.e., below 0.5–0.6 T_melt_. This phenomenon has been observed in a number of UFG Al alloys, where a special state of grain boundaries is formed. This involves the formation of segregations of individual alloying elements, or even layers of new phases along the grain boundaries. The formation of such grain boundaries ensures the manifestation of the SP effect in these alloys at unusually low temperatures, and in some cases even at room temperature.

At the same time, the formation of nanostructures with grain boundary precipitates in the form of segregations of atoms of certain alloying elements or nano-sized particles of the second phases of a given composition and morphology can be achieved through the use of specially selected regimes and schemes of thermomechanical processing, including SPD.

The role of specific alloying elements in the manifestation of low-temperature superplasticity is of significant importance. For instance, the addition of Zn to Al alloys with ultrafine grains enables a significant reorganization of high-angle boundaries. This is achieved through accelerated diffusion processes, which redistribute Zn atoms during deformation and result in the formation of segregations or nanolayers along grain boundaries, facilitating grain boundary sliding. At that time, alloying Mg and a number of other elements introduces complications to the deformation mechanisms, leading to low-temperature SP. To gain a deeper understanding of this phenomenon, further studies of the structure and properties of grain boundaries are required.

At the same time, the discovery of the low-temperature superplastic behavior of Al alloys with ultrafine grains permits the development of efficient technologies to build products with complex geometrical shapes from lightweight materials to be used in engineering structures where weight minimization and high strength are crucial, since the preservation of the UFG structure after low-temperature deformation ensures that such materials retain very high strength.

This problem was recently addressed in detail in the work [28], where the authors investigated the UFG alloy Al-Zn-Mg-Zr processed by SPD, which exhibited SP behavior at unusually low temperatures of 120–170 °C, while maintaining a high-strength state at room temperature with an ultimate tensile strength 25–50% higher than after standard T6 treatment. The analysis revealed that the high-strength state is primarily due to the preservation of a stable UFG structure in the alloy, even after a significant degree of SP deformation. These results indicate a significant innovative potential of low-temperature superplasticity in UFG alloys and open prospects for the comprehensive development of SP forming of complex-profile products.

The maintenance of ultrafine-grained structure after low-temperature SP deformation ensures the retention of exceptionally high strength in these materials. Another promising approach is the manufacturing of metal–matrix composites by the solid-phase method, which involves the pressing of reinforcing elements and matrix. In a recent study [40], it was demonstrated that the production of metal–matrix composites from superplastic Al matrices of 1565 alloys of the Al-Mg system in ultrafine-grained states and long-sized boron fibers as reinforcing materials is possible when low values of applied stresses are employed, and relatively low deformation temperatures of 300 °C are achieved. This results in the formation of chemical interaction products at the “fiber–matrix” interface, which ensures the integrity of the fibers. This method allows the formation of a composite material with high physical and mechanical properties.

In general, the results of recent studies on low-temperature superplasticity in Al alloys, as presented herein, clearly illustrate both the fundamental advances associated with achieving unusual mechanical properties in materials through engineering their UFG structure and grain boundary behavior, as well as the great innovative potential of this approach, which has the potential to improve the efficiency of superplastic forming of complex-shaped products and to facilitate the development of advanced metal matrix composites.

## Figures and Tables

**Table 1:** Summary of representative low temperature superplasticity reports in aluminum alloys.

Alloy	Processing	Grain Size (nm)	Elongation (%)	Temperature (°C)	Strain Rate (s^−1^)	m	[Ref.]
7075Al	FSP	0.8	350	200	1 × 10^−5^	0.36	[13]
Al–4Mg–1Zr	FSP	0.7	240	175	1 × 10^−4^	0.34	[14]
Al–Zn–Mg–Sc	FSP	0.68	525	220	1 × 10^−2^	0.33	[18]
5083Al	ECAP	0.3	315	275	5 × 10^−4^	0.4	[19]
7055Al	ECAP	1	320	300	5.6 × 10^−5^	0.34	[20]
A7075	HPT	120	760	350	7.0 × 10^−3^	0.3	[15]
Al-Zn-Mg-Zr	HPT	100	500	170	5 × 10^−4^	0.43	[23]
Al-7Mg	ECAP	0.5	500	300	10^−3^	0.75	[21]
5083Al	ARB	0.28	230	200	1.7 × 10^−3^	0.37	[12]
AA5083	ECAP	0.5		300	3 × 10^−3^	0.3	[22]
